# Increasing Boiling Heat Transfer using Low Conductivity Materials

**DOI:** 10.1038/srep13145

**Published:** 2015-08-18

**Authors:** Md Mahamudur Rahman, Jordan Pollack, Matthew McCarthy

**Affiliations:** 1 Department of Mechanical Engineering and Mechanics, Drexel University, Philadelphia, PA, USA

## Abstract

We report the counterintuitive mechanism of increasing boiling heat transfer by incorporating low-conductivity materials at the interface between the surface and fluid. By embedding an array of non-conductive lines into a high-conductivity substrate, in-plane variations in the local surface temperature are created. During boiling the surface temperature varies spatially across the substrate, alternating between high and low values, and promotes the organization of distinct liquid and vapor flows. By systematically tuning the peak-to-peak wavelength of this spatial temperature variation, a resonance-like effect is seen at a value equal to the capillary length of the fluid. Replacing ~18% of the surface with a non-conductive epoxy results in a greater than 5x increase in heat transfer rate at a given superheat temperature. This drastic and counterintuitive increase is shown to be due to optimized bubble dynamics, where ordered pathways allow for efficient removal of vapor and the return of replenishing liquid. The use of engineered thermal gradients represents a potentially disruptive approach to create high-efficiency and high-heat-flux boiling surfaces which are naturally insensitive to fouling and degradation as compared to other approaches.

Boiling heat transfer is used in many applications, including the production of electricity, chemical processing, and high-heat flux thermal management. Due to its prevalence, and the important role it plays in numerous industries, the enhancement of boiling heat transfer has been studied for decades. During boiling, heat is transferred from a hot surface to a cooler fluid by (1) evaporation across liquid-vapor interfaces, (2) transient conduction, and (3) the micro-convection generated by the nucleation, growth, and departure of vapor bubbles^1^,^2^. The efficiency of boiling is quantified by the heat transfer coefficient (HTC), defined as the ratio of the surface heat flux to the superheat temperature, the temperature difference between the solid and the saturated fluid. During boiling, there is a finite rate at which heat can be dissipated from any surface. This maximum is the critical heat flux (CHF). CHF, also referred to as the boiling crisis, occurs when the production of vapor cannot be adequately balanced by the amount of liquid returning to the heated surface. When this occurs the surface undergoes dry-out, where an insulating layer of vapor blankets the solid surface. This leads to an immediate, uncontrollable, and drastic increase in surface temperature with dangerous and potentially catastrophic consequences, such as the destruction of electronic components or the meltdown of a nuclear reactor.

Understanding and improving HTC and CHF in boiling systems has been extensively studied for decades, including the development of a variety of enhancement strategies^3^,^4^. Traditionally, the use of enhanced surfaces has been primarily focused on the creation of structures and complex geometries machined into, or attached onto, the boiling surface such as porous materials, sintered wires and meshes, as well as mechanically deformed structures^5^,^6^,^7^. With the development of increasingly precise fabrication tools, micro/nano-structured surface coatings have been explored in recent years, showing substantial increases in boiling performance. A variety of microscale and nanoscale wires, rods, posts, and other structures have been fabricated on silicon chips and shown to enhance HTC and CHF of water anywhere from 50% to 300%, depending on the working fluid^8^,^9^,^10^,^11^,^12^,^13^,^14^,^15^,^16^. Similarly, micro/nano-structures on copper and other metals have also been demonstrated^1^,^17^,^18^,^19^. The role that surface structures play in boiling enhancement has been attributed to a variety of factors, including capillary wicking^9^,^12^,^13^,^14^,^17^, increased nucleation site densities^9^,^20^, and increased contact line pinning^10^. Additionally, notable increases in HTC have been reported using embossed copper microstructures with specific contoured shapes^1^. These complex microscale shapes use evaporation momentum force to promote the separation of liquid and vapor flow paths and enhance micro-convection.

While structured surfaces have been shown to greatly increase boiling heat transfer, the reliability of these approaches for real-world applications is in question due to a variety of factors. All structured surfaces are inherently susceptible to mechanical failure (breaking of the micro/nano-structures), as well as fouling and clogging over time. During boiling, any and all contaminants within the fluid will inevitably be drawn into the structures. This will lead to clogging and filling of the small micro/nano-scale voids, and thus a loss of enhancement^19^. Similarly, the robustness of extremely thin nanostructured coatings (≤1 μm) has not yet been demonstrated, leaving the potential for the coatings to be destroyed or altered over time via chemical reactions^21^. The use of low-surface-energy materials to promote nucleation at small superheats has been successfully demonstrated as well^22^,^23^,^24^,^25^, however these biphilic surfaces are prone to degradation of the thin non-wetting films. Additionally, they are not effective with all working fluids, in particular highly wetting fluids like FC-72. While doubly reentrant surfaces have been demonstrated to repel these highly wetting fluids^26^, their use in biphilic designs for boiling enhancement has not yet been demonstrated.

These enhancement techniques rely on surface properties and interfacial phenomena, which are inherently susceptible to degradation. Here we report the enhancement of CHF and HTC using surfaces with in-plane variations in substrate thermal conductivity. These “bi-conductive” surfaces are comprised of rows of low-conductivity epoxy embedded into high-conductivity copper substrates, and therefore rely on bulk properties rather than surface properties to enhance boiling. They create spatial variations in the surface temperature during boiling, and by tuning the wavelength of these variations to coincide with the capillary length, have shown increases in HTC and CHF of greater than a factor 5x and 2x, respectively. These counterintuitive results are explained by an examination of the resulting liquid and vapor flow fields, as well as enhanced bubble dynamics. This novel enhancement mechanism has not yet been observed or studied, and holds the potential for creating robust and reliable surfaces for high-efficiency and high heat flux boiling applications over long lifetimes.

## Results

### Bi-conductive surfaces for tailoring bubble dynamics during boiling

**Figure 1:**
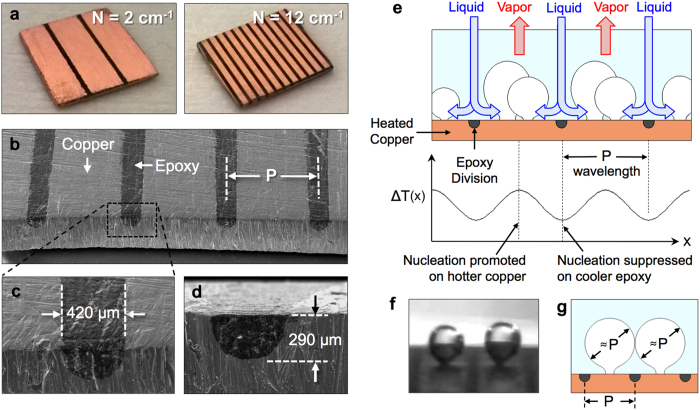
Bi-conductive surfaces to tailor spatial variations in wall temperature. The fabricated surfaces showing (**a**) samples with two and twelve divisions per centimeter, and (**b–d**) scanning electron microscope (SEM) images of the low-conductivity epoxy divisions embedded in high-conductivity copper. (**e**) Schematic of the resulting flow field where spatial variations in wall superheat temperature, ΔT(x), promotes spatial ordering. Nucleation sites, as well as the liquid and vapor flow paths, are tailored to coincide with the wavelength of the in-plane temperature variations given by the pitch, P. (**f**) Image of the onset of nucleate boiling (ΔT ~ 5 K), where bubbles preferentially nucleate in the center of the copper segments resulting in (**g**) control over the nominal bubble diameter during lateral coalescence.

Figure 1a–d shows the bi-conductive surfaces fabricated and tested for the current work. Grooves were first machined into copper substrates and subsequently filled with a two-part high-temperature epoxy. After curing the entire part was sanded by hand to remove excess epoxy, resulting in flat surfaces with lines of epoxy dividing the copper into parallel strips. Bi-conductive surfaces were fabricated with a varying number of epoxy divisions per centimeter, *N*. Figure 1a shows optical images of the *N* = 2 cm^−1^ and *N* = 12 cm^−1^ designs. In total, seven distinct surfaces have been fabricated and tested including a bare copper surface and bi-conductive surfaces with *N* = 2, 4, 6, 8, 10, and 12 cm^−1^. Figure 1b–d shows scanning electron microscope (SEM) images of the embedded epoxy divisions which have a measured width of 420 ± 5 μm, depth of 290 ± 23 μm, and center-to-center pitches of P = 0.96–3.7 mm.

The bi-conductive surfaces have a periodic arrangement of low-conductivity material embedded within a high-conductivity substrate. The thermal conductivity of the epoxy (<1 W/mK) is several orders of magnitude less than that of copper (~400 W/mK). When submerged in a fluid and heated from the underside, this creates an in-plane variation in surface temperature. Due to the extreme conductivity difference, there is negligible heat conducted through the epoxy divisions during nucleate boiling (see thermal circuit analysis in the Supplementary Material). As such, the wall superheat temperature above the epoxy divisions will be notably reduced as compared to that experienced over the copper sections. At low average wall superheats, prior to bubble nucleation, these in-plane temperature variations will drive natural convection flows aligned with the epoxy divisions similar to Rayleigh-Bérnard convection rolls. It has been shown that this sort of flow instability is immediately triggered for any finite superheating with a spatially varying surface temperature^27^. The resulting flow-field draws cooler liquid down toward the surface from above the epoxy divisions, while warmer fluid rises from above the hot copper. At these early stages the counter-rotating flows will produce a stagnation point in the center of the copper, resulting in a local minimum in convective heat transfer coefficient and therefore a local maximum in surface temperature^28^. As the wall superheat is increased this local maximum temperature promotes bubble nucleation along the center of the copper regions. This results in the formation of an ordered flow-field, as shown schematically in Fig. 1e, which persists at increasing thermal loadings. The in-plane variations in wall superheat take a periodic form with a wavelength equal to the pitch between epoxy divisions, *P*. This superheat wavelength dictates the nature and location of the ordered pathways of escaping vapor and replenishing liquid returning to the surface during nucleate boiling. Figure 1f shows an image of the onset of nucleate boiling, where the first few nucleation sites are activated on a bi-conductive surface (*N* = 4 cm^−1^) at a wall superheat of ~5 K. As can be seen, the vapor bubbles preferentially nucleate near the center of the copper due to the local maximum in surface temperature. This is consistently seen for all of the surfaces tested, and demonstrates that the wettability of the epoxy is not promoting nucleation due to a lower surface energy (see supplemental movies). Additionally, Fig. 1g shows how the superheat wavelength, *P*, imparts a level of control over the bubble diameter during lateral coalescence. By ordering nucleation sites and liquid return pathways, the size of bubbles undergoing lateral coalescence across an epoxy division is tuned to nominally coincide with the superheat wavelength, *P*.

These bi-conductive surfaces have been shown here to promote HTC and CHF by ordering the vapor and liquid flow-fields during pool boiling, resulting in enhanced bubble dynamics and departure, as well as a delay of the boiling crisis. These enhancements are explicitly derived from bulk material properties (thermal conductivity), as opposed to surface properties or surface structures. Unlike surface coatings, this fundamental mechanism is not susceptible to material degradation and log-term reliability issues. Nor is it reliant on specific fluids or fluid properties to promote boiling, as is required for surfaces incorporating low surface energy materials. Similarly, these flat bi-conductive surfaces do not rely on surface wicking nor are they susceptible to mechanical failure or clogging, which will occur on structured surfaces. Additionally, they can be easily fabricated on a variety of materials using traditional machining techniques and do not rely on complex manufacturing, or precise geometric shapes, to impart boiling enhancement.

### Pool boiling characterization and high-speed imaging

Each of the fabricated surfaces has been characterized during pool boiling of saturated water at atmospheric conditions using a custom built experimental apparatus. The complete details of the apparatus, testing procedures, and experimental uncertainty can be found in the Methods Section as well as prior publications^14^,^19^. Briefly, each surface was soldered to an insulated copper heater block and submerged in a bath of saturated water. The heat flux delivered to the surface, *q”*, is periodically increased in small increments and the system is left to reach thermal equilibrium, typically 10–20 minutes. After this the surface temperature and heat flux are recorded, and the process is repeated up until the CHF is reached. CHF is taken as the highest stable heat flux prior to the uncontrollable increase in surface temperature associated with dry-out. The average wall temperature at the surface of the sample, 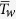, is measured using a thermocouple placed at the top of the heater block, and accounts for the various thermal resistances between this thermocouple and the surface. These include the resistances across the solder interface and bulk copper^14^,^19^, as well as the resistance across the composite copper-epoxy region near the surface of the sample. While the embedded epoxy produces in-plane temperature variations on the surface, it does not greatly impact the overall thermal resistance of the sample itself. For the extremely thin samples tested in this work (1 mm thickness), the increase in overall thermal resistance of the substrate was ~6% for the *N* = 4 cm^−1^ design. For a more realistic wall thickness of 1/4 inch (6.35 mm), the increase in thermal resistance associated with adding embedded epoxy divisions drops to less than 1%.

During each test the average wall superheat, Δ*T*, is measured where


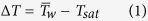


and the associated heat transfer coefficient (HTC) is calculated as


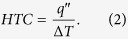


Additionally, visualization of the boiling process is conducted using a Phantom V210 high-speed camera (Vision Research) recorded at over 3,000 fps. High-speed imaging is used to visualize bubble dynamics during nucleate boiling at low heat fluxes. At higher fluxes the large production of vapor makes visualization difficult, resulting in no useful information regarding the impact of bi-conductive surface on the boiling process.

**Figure 2:**
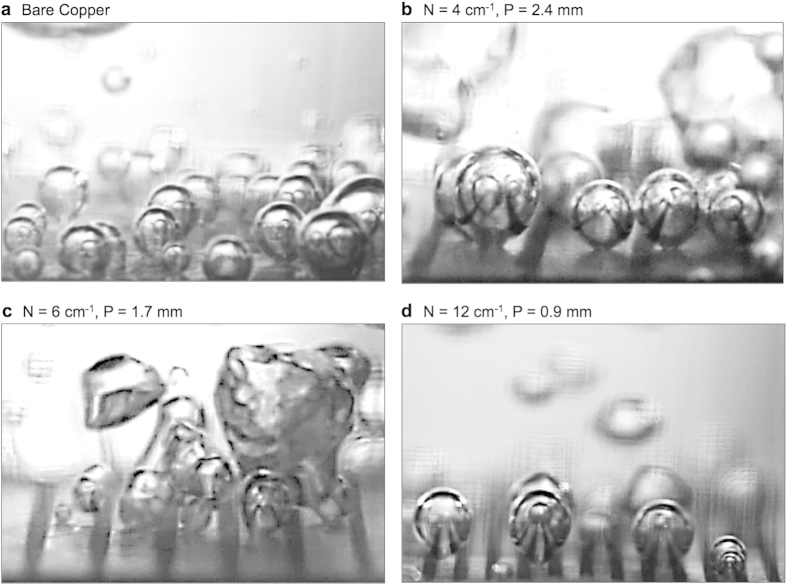
Nucleate boiling on bi-conductive surfaces. High-speed imaging of nucleate boiling at ~10–15 W/cm^2^ on (**a**) a bare copper surface and bi-conductive surfaces with (**b**) *N* = 4 cm^−1^, (**c**) *N* = 6 cm^−1^, and (**d**) *N* = 12 cm^−1^ designs. For each bi-conductive surface, nucleation is seen in the center of the high-temperature copper and no lateral motion of the three-phase contact lines beneath vapor bubbles is observed. The low-temperature epoxy divisions remain wetted at all times providing ordered pathways near the surface for replenishing liquid to feed the bubble ebullition cycles. See supplementary information for complete movies.

**Figure 3:**
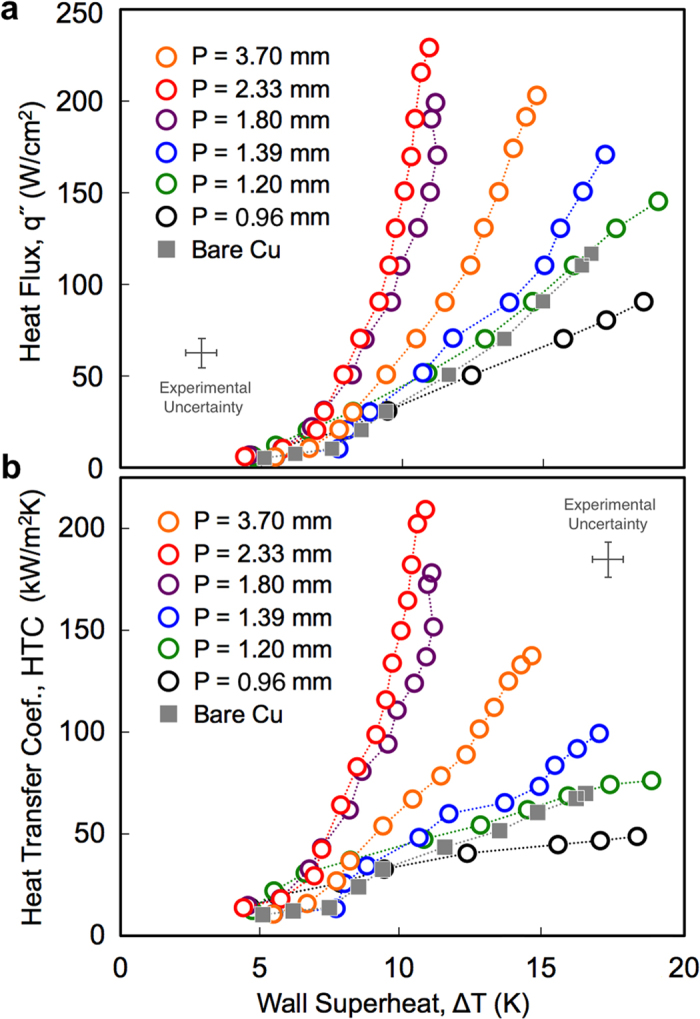
Boiling curves and heat transfer coefficient. Experimental results for (**a**) heat flux and (**b**) heat transfer coefficient as a function of wall superheat temperature. Increased performance is seen for five of the six bi-conductive surfaces, corresponding to those with wavelengths of P = 1.2–3.7 mm. A decrease in performance is seen for the P = 0.96 mm design.

Figure 2 shows representative images extracted from high-speed imaging, showing nucleate boiling on bi-conductive surfaces with various values of *N*. Complete movies can be found in the supplemental material. All of the surfaces tested exhibited similar incipience temperatures, with the onset of nucleate boiling occurring around Δ*T *~ 5–7 K. It can be seen that bubbles form exclusively on the copper regions, with the low-temperature epoxy divisions suppressing the nucleation process as expected. More importantly, the epoxy divisions remain wetted at all times and resist the lateral motion of bubbles across them. This allows for the formation of ordered pathways for liquid return above the cold epoxy divisions. Figure 3 shows the measured boiling curves, and heat transfer coefficient as a function of superheat for each surface tested. The bare copper surface reached a CHF of ~116 W/cm^2^ with a maximum HTC of ~70 kW/m^2^K. This closely matches the results from various other researchers, demonstrating the accuracy of the experimental apparatus^1^,^11^,^20^. Substantial increases in boiling performance can be seen for nearly all of the bi-conductive surfaces as compared to bare copper. At moderate superheats (Δ*T* = 10–11 K), an increase in heat flux and heat transfer coefficient of greater than 5x has been demonstrated using bi-conductive surfaces with a pitch of P = 2.33 mm (*N* = 4 cm^−1^). While the incipience temperature does not vary substantially for any of the surfaces tested, heat transfer is increased due to enhanced bubble dynamics on the bi-conductive surfaces. This demonstrates that the addition of epoxy does not enhance HTC by promoting nucleation at lower superheats, as shown for structured surfaces and surfaces with mixed wettability^1^,^9^,^11^,^20^,^22^,^23^,^25^.

By creating in-plane variations in the superheat temperature, the nucleation, growth, and departure of vapor has been substantially affected. Spatial ordering of the flow field allows for the efficient removal of vapor and return of liquid, accelerating the ebullition cycle and promoting the departure of bubbles. The efficient return of replenishing liquid has been seen to delay dry-out and enhance CHF up to a factor of 2x using bi-conductive surfaces. While no formal characterization of the long-term reliability of the samples has been conducted, none of the bi-conductive surfaces tested in this work have shown any mechanical failure or degradation during several hours of testing.

## Discussion

**Figure 4:**
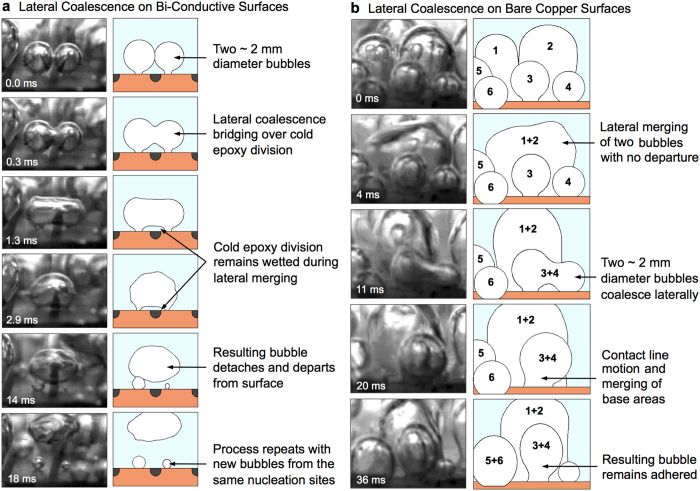
Lateral coalescence, contact line motion, and bubble departure. High-speed time lapse imaging of the lateral coalescence of attached vapor bubbles on (**a**) bi-conductive surfaces, and (**b**) bare copper surfaces at ~10–15 W/cm^2^. (**a**) When two bubbles laterally coalesce over an epoxy division, the cold epoxy remains wetted and no contact line motion is observed. By remaining wetted at all times, the epoxy divisions promote departure at small diameters and maintain ordered flow paths for replenishing liquid. (**b**) Conversely, lateral coalescence on bare copper surfaces typically includes the lateral motion of contact lines, and merging of non-wetted base areas beneath the vapor bubbles.

The enhancements shown here have been attributed to the ability of in-plane variations in wall superheat to impart order to the resulting liquid and vapor flows, leading to high HTC and a delay in CHF. By reducing the superheat temperature in the vicinity of the low-conductivity materials, the epoxy divisions suppress nucleation and remain wetted during boiling. While this reduces the local heat transfer rate near the epoxy, it increases the global heat transfer rate over the entire surface by imparting spatial ordering and enhanced bubble dynamics. For this order to be maintained the cold epoxy divisions must remain wetted at all times. Figure 4 shows time-lapse imaging of vapor bubbles undergoing lateral coalescence, and demonstrates the ability of the epoxy divisions to remain wetted during boiling. See supplementary information for complete movies.

In all of the imaging of bare copper surfaces carried out in this work, the three-phase contact line beneath a bubble undergoes visible in-plane motion during lateral coalescence, as is typically seen^29^,^30^. This behavior, however, is not seen on the bi-conductive surfaces where the epoxy divisions remain wetted at all times and effectively create boundaries that the three-phase contact lines do not move over. This behavior is essential for creating and maintaining ordered pathways of escaping vapor and replenishing liquid and is seen as the key contributor to boiling enhancement. Several researchers have observed and characterized the complex motion of contact lines during nucleate boiling and boiling crisis. These include the work by Kim *et al*.^31^ and Jung *et al*.^32^ using IR thermometry to visualize the liquid-solid interface and measure contact line length, density, and speed.

In addition to spatially ordering the escaping vapor and returning liquid flows, the epoxy divisions promote the coalescence-induced departure of laterally merging bubbles. Figure 4a shows the coalescence of two ~2 mm bubbles bridging over an epoxy division. As the two bubbles merge the underlying epoxy division between them remains wetted at all times. A thin liquid layer remains on the cold epoxy surface and the non-wetted base areas below each bubble do not merge, collapse, or otherwise move during the entire process. The resulting bubble deforms due to surface tension and is quickly ejected from the surface. The low temperature epoxy inhibits the inward lateral merging of the non-wetted bases, and helps draw replenishing liquid underneath the bubble thus promoting departure. Conversely, Fig. 4b shows time lapse imaging during pool boiling on bare copper surfaces at a comparable heat flux, where three distinct lateral coalescence events are visible. During the 36 ms movie several bubbles are seen coalescing laterally, and in each event the non-wetted base areas merge inward and the resulting bubbles do not depart. The result is a new non-wetted base area beneath the larger coalesced bubble that is still attached to the surface. While this high-speed imaging is conducted at relatively low heat fluxes, it does provide insight into the mechanism driving boiling enhancement on bi-conductive surfaces. The behaviors visualized at low fluxes, along with the pool boiling results shown in Fig. 3, can be used to draw conclusions regarding the nature of enhancement across all boiling regimes up to CHF.

**Figure 5:**
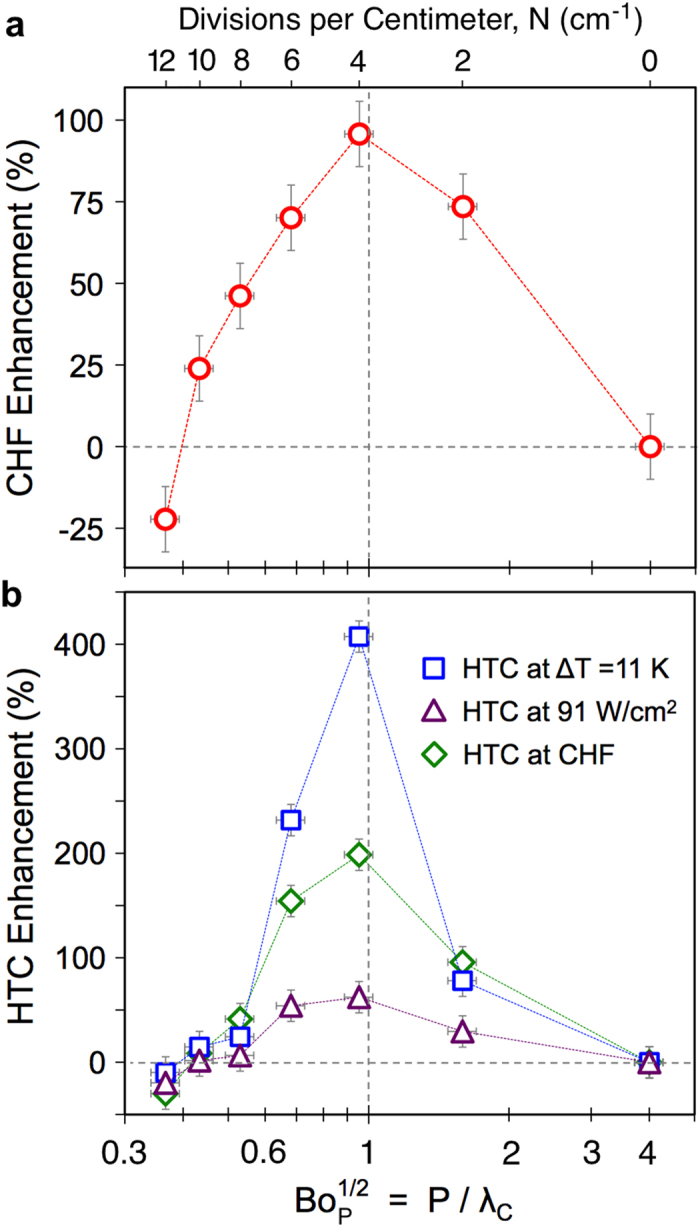
Boiling enhancement and the effect of Bond number. Experimental results showing the percent increase in (**a**) critical heat flux (CHF), and (**b**) heat transfer coefficient (HTC) for bi-conductive surfaces as compared to bare copper surfaces. Optimal performance is seen at Bond numbers of unity with a 2x increase in CHF and a 5x increase in HTC. These results highlights the importance of tuning the wavelength of spatial temperature variations, *P*, with the capillary length of the working fluid, *λ*_*C*_, resulting in enhanced boiling performance over all stages of boiling.

The enhancement mechanisms proposed here are inherently linked to the wavelength of the spatial variations in surface superheat, *P*, as illustrated in Fig. 1. To probe the effect of this wavelength on boiling performance, the pitch between epoxy divisions has been systematically varied. The percent increase in CHF and HTC of the bi-conductive surfaces relative to bare copper is plotted in Fig. 5 against the superheat wavelength, *P*, normalized by the capillary length, *λ*_C_. The capillary length is the characteristic length scale of an interface subject to both surface tension and gravitational forces, and for a bubble is given by


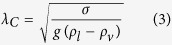


where *σ* is the surface tension at the interface, *g* is the acceleration of gravity, and *ρ*_*l*_ and *ρ*_*v*_ are the densities of the liquid and vapor phases, respectively. The relative importance of surface tension and buoyancy (gravitational) forces on a bubble is captured through the nondimensional Bond number, Bo, given by


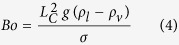


where *L*_c_ is the characteristic length. Defining the Bond number using the wavelength of the superheat variations, *P*, as the characteristic length of the surface results in


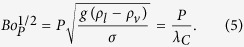


Figure 5 shows the importance of Bond number and capillary length on the boiling enhancement of bi-conductive surfaces, where all fluid properties are evaluated at the saturation temperature and the edge length of the sample is used as the characteristic length for bare copper. A clear maximum in performance is seen when the superheat wavelength (and pitch between epoxy divisions, *P*) coincides with the capillary length of the fluid, and the Bond number approaches unity. This optimal wavelength can be explained by examining the nominal bubble departure diameter during boiling. This is traditionally characterized by defining the Bond number using the bubble departure diameter, *D*, as the characteristic length. Numerous correlations exist to predict bubble departure using the nondimensional departure diameter given by 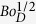
33,34,35,36,37. These include the classical works of Fritz^36^ and Cole and Rohsenow^34^, which have shown good agreement with experimental results for decades. Fritz’s empirical correlation relates the departure diameter to the contact angle of the surface, θ, and is given by


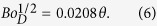


Cole and Rohsenow’s correlation for water is given by


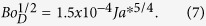


The effects of sensible and latent heat are incorporated in this correlation through the use of the modified Jakob number defined as


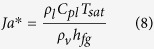


where *C*_*pl*_ is the heat capacity of the liquid and *h*_*fg*_ is the latent heat of vaporization^29^. Evaluating equations (6) and (7) for saturated water on copper results in values of 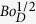 = 1.04 ± 0.1 and 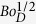 = 0.98 ± 0.1, respectively, considering the uncertainties in contact angle and material properties. The bubble departure diameters predicted by both models closely match the optimal pitch between epoxy divisions found experimentally to be 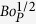 ≈ 1.

These predictions show that as the wavelength of the variations in superheat, *P*, approaches the departure diameter of the bubbles, a resonance-like effect is seen leading to substantial increases in both HTC and CHF. Interestingly, Fig. 5b shows that this resonance-like enhancement in heat transfer is seen at the same wavelength for all phases of boiling. At a common wall superheat of Δ*T* = 11 K, the heat transfer rates of the various surfaces range from 40–230 W/cm^−2^ (Fig. 3a). This corresponds to a greater than 5x difference in vapor production rates between the highest and lowest performing surfaces. At a common heat flux of 91 W/cm^−2^, the required wall superheat more than doubles across the surfaces tested, varying from 9 K to 19 K. At CHF, each surface is operating at distinctly different heat fluxes (from 91 to 230 W/cm^−2^) and wall superheats (from 11 to 19 K). Yet in all three cases, the maximum HTC is observed for bi-conductive surfaces where the pitch between epoxy divisions, *P*, is equal to the capillary length, *λ*_C_.

Adding epoxy divisions and therefore reducing the area available for heat transfer by ~18%, results in up to a 5x increase in heat transfer rate at a given superheat. This result is explained by examining the nature of the bubble nucleation, growth, and departure process. The addition of non-conductive epoxy imparts order to the resulting vapor and liquid flows, which helps draw cool liquid to the surface and promotes departure of nucleating bubbles. While less of the surface area is available to transfer heat, this spatial order enhances the ebullition cycle and also delays dry-out. Figure 5 shows with the addition of two and four epoxy divisions per centimeter (*N* = 2 cm^−1^ and *N* = 4 cm^−1^ designs), the heat transfer rates steadily increase as compared to the bare surface. The performance enhancement reaches a maximum at a Bond number of unity, 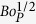 = 1, and then decreases for smaller Bond numbers (*N* ≥ 6 cm^−1^). As the Bond number decreases, two factors affecting performance arise. First, the pitch between the epoxy divisions becomes smaller than the nominal bubble departure diameter, and secondly, the effect of surface tension becomes more pronounced. For *N* ≥ 6 cm^−1^ bubbles grow to diameters wider than the pitch between epoxy divisions before they can depart. This impedes the counter flow of cooler liquid returning to the surface and disrupts the desired spatial ordering of flow fields. Additionally, for *Bo*_P_ < 1 the effects of surface tension on the bubble departure process is evident, where the non-wetted base area beneath a bubble can grow to the entire width of the copper section (see supplementary movies). This is particularly evident for the *N* = 12 cm^−1^ design, which has a Bond number of *Bo*_P_ = 0.13, and shows performance worse than bare copper. In this case, the reduction of area available to conduct heat and the adverse effects of surface tension have outweighed any potential improvements associated with ordered flows. A decrease in HTC and CHF of approximately 25% as compared to a bare copper surface is seen.

This work has shown that by imparting in-plane variations in surface temperature onto flat surfaces using low-conductivity materials, extreme increases in pool boiling heat transfer can be achieved. By tuning the wavelength of these variations to coincide with the capillary length of the fluid, a resonance-like enhancement effect is seen leading to a greater than 5x improvement in heat transfer rate at moderate superheats. The counterintuitive result by which heat transfer is increased with the addition of non-conductive materials is shown to be a product of the resulting flow field near the surface. By using temperature gradients to order the location of nucleation sites, as well as promote the formation of distinct pathways for liquid and vapor flows, HTC has been increased from 41 kW/m^2^K to 210 kW/m^2^K (at Δ*T* = 11 K) and CHF has been increased from 116 W/cm^2^ to 230 W/cm^2^. The principle of tailoring flow fields through variations in local surface temperature represents a novel and potentially transformative tool for the enhancement of boiling heat transfer in next-generation high heat flux applications. Bi-conductive surfaces not only produce substantial increases in performance (>5x increase in HTC shown here), but are also cheaply manufactured using traditional methods, scalable to large areas and various material, and do not contain fragile surface structures or thin coatings. As a result they are easily implementable and naturally insensitive to the degradation, mechanical failure, and fouling associated with other boiling enhancement approaches.

## Methods

### Bi-Conductive Surface Fabrication

Bi-conductive surfaces were fabricated by embedding lines of a low-conductivity epoxy into a copper substrate. Copper sheets (1 mm thickness) were cut to size and grooves were machined into them using Wire Electrical Discharge Machining (EDM). The EDM wire thickness was 0.254 mm with a reported minimum spark gap of 0.381 mm ± 0.127 mm. The copper was then treated with an alkaline solution to produce an oxide layer with nano-scale surface roughness to promote adhesion between the copper and epoxy^14^. The surfaces were then coated with a non-conductive high-temperature two-part epoxy (Aremco 526N) filling all of the grooves. The epoxy was cured at 93 °C for 2 hours, followed by 163 °C for 12 hours to achieve a maximum strength bond. After curing, the surfaces were manually sanded with 200 grit sand paper until the bare copper between each epoxy division was exposed. The bare copper surfaces (with no epoxy divisions) were also sanded using the same method. The surfaces were finally cleaned with solvents and dried with N_2_.

### Pool Boiling Characterization and Imaging

Surfaces were characterized using a custom-built test set-up as previously reported by Rahman *et al*.^14^,^19^. The setup consists of a copper heater block with PTFE insulation embedded with two cartridge heaters allowing for a maximum power of 1,000 W. Five T-type thermocouples were inserted into the copper block equally spaced 6 mm apart with the top most thermocouple located directly beneath the sample. The temperature measurements were recorded using NI DAQ system, where the average heat flux in the copper block was calculated using Fourier’s conduction law. The sample surface temperature was calculated by considering all of the relevant thermal resistances between the surface and the top most thermocouple, as described. A polycarbonate chamber was used as the water bath; with an immersion heater and thermocouple probe maintaining saturated conditions and atmospheric pressure. Degassed, deionized water was used as the working fluid, and all tests were carried out up to CHF. Visualization of the boiling process was conducted at low heat fluxes using a Phantom V210 high-speed camera (Vision Research) recorded at 3,100 fps. The surfaces were initially maintained at saturated condition for 1 hour after which a small increment of heat flux (2 W/cm^2^ to 5 W/cm^2^) was applied to the surfaces until the nucleation was observed. Heat flux was further increased up to 20 W/cm^2^ until the visibility of bubble dynamics becomes difficult. The chamber heater was turned off to minimize the bulk fluid motion while capturing the movies, with all movies being recorded within one minute to maintain saturation conditions, as described by Mukherjee and Dhir^30^ and Son *et al*.^38^.

## Additional Information

**How to cite this article**: Rahman, M.M. *et al*. Increasing Boiling Heat Transfer using Low Conductivity Materials. *Sci. Rep*. **5**, 13145; doi: 10.1038/srep13145 (2015).

## Supplementary Material

Supplementary Information

Supplementary Movie S1

Supplementary Movie S2

Supplementary Movie S3
