# Supplementary material for: Increasing Boiling Heat Transfer using Low Conductivity Materials
Source: Sci Rep. 2015 Aug 18;5:13145. doi: 10.1038/srep13145 (PMC4539544; doi:10.1038/srep13145)
Supplement: Supplementary Information [file srep13145-s1.pdf]

Supplementary Information

INCREASING BOILING HEAT TRANSFER USING LOW CONDUCTIVITY MATERIALS

Md Mahamudur Rahman, Jordan Pollack, and Matthew McCarthy*

Department of Mechanical Engineering and Mechanics, Drexel University,
3141 Chestnut St, Philadelphia, PA, 19104 USA

*email: mccarthy@coe.drexel.edu

1. Supplemental Movies

Movie #1: Preferential Nucleation

Showing the preferential nucleation of vapor bubbles along the copper areas, as opposed to the low-temperature epoxy divisions on a bi-conductive surface with $N = 4$ cm^{-1}. The movie is recorded at 3,100 frames per second and played back at 31 frames per second (100 times reduced speed).

Movie #2: Nucleate Boiling

Showing nucleate boiling on a bare copper surface and three bi-conductive surface corresponding to $N = 4$ cm^{-1}, $N = 6$ cm^{-1}, and $N = 12$ cm^{-1} designs. The movies are recorded at 3,100 frames per second and played back at 31 frames per second (100 times reduced speed).

Movie #3: Lateral Coalescence

Showing the lateral coalescence of select vapor bubbles on a bare copper surface and a bi-conductive surface with $N = 4$ cm^{-1}. The movie is recorded at 3,100 frames per second and played back at 31 frames per second (100 times reduced speed).

2. Thermal Circuit Analysis

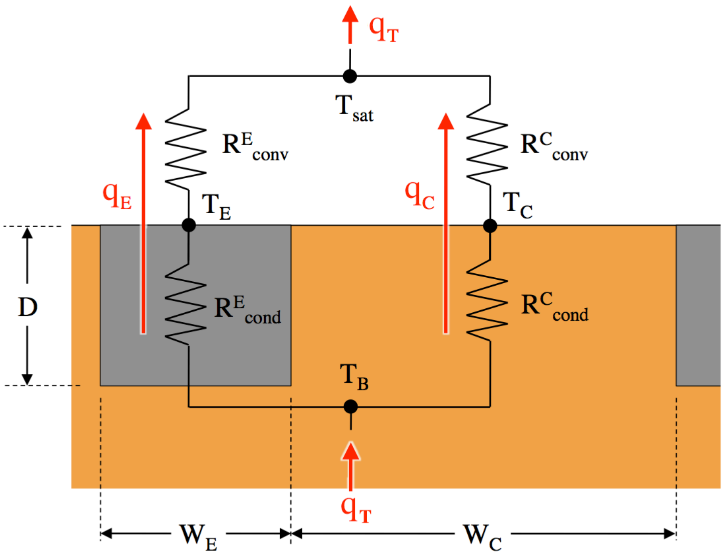

$$R^E_{conv} = \frac{1}{h\,A_E} = \frac{1}{h\,W_E L} \quad \text{(S1)}$$

$$R^C_{conv} = \frac{1}{h\,A_C} = \frac{1}{h\,W_C L} \quad \text{(S2)}$$

$$R^E_{cond} = \frac{D}{k_E\,A_E} = \frac{D}{k_E\,W_E L} \quad \text{(S3)}$$

$$R^C_{cond} = \frac{D}{k_C\,A_C} = \frac{D}{k_C\,W_C L} \quad \text{(S4)}$$

Figure S1: *Thermal circuit diagram for a segment of a bi-conductive surface showing the relevant conductive and convective resistances between the base copper and the saturated fluid.*

To understand the variations in surface temperature and the relative rates of heat transfer through the epoxy and copper sections of the bi-conductive surfaces, a first order thermal circuit analysis has been conducted. Figure S1 shows a segment of a bi-conductive surface with an embedded epoxy division approximated with a rectangular cross

section. The epoxy dimensions are W_E = 0.42 mm and D = 0.29 mm, as measured in SEM imaging (Figure 1). The width of the copper section is given by $W_C = P - W_E$ where the pitch P varies for each surface from 0.96 to 3.7 mm. This results in copper widths of W_C = 0.54 – 3.28 mm for the six bi-conductive surfaces fabricated and tested.

Equations S1 and S2 give the convective thermal resistances in the water above the epoxy and copper sections, while Equations S3 and S4 give the conductive resistances through the epoxy and copper. In Equations S1-S4, k is the thermal conductivity, L is the length of the epoxy division (also the edge length of the sample), and the subscripts E and C are used to denote the epoxy and copper, respectively. It is assumed in this analysis that k_C = 400 W/mK and k_E = 1 W/mK. The heat transfer coefficient in the fluid is given by h, and assumed to be constant across the whole surface. The total heat transfer rate passing through this segment of the bi-conductive surface is given by q_T, with some portion of that passing through the epoxy (q_E) and some passing through the copper (q_C), such that

$$q_T = q_E + q_C . \qquad \text{(S5)}$$

Using this thermal circuit model, the fraction of the heat transfer passing through the epoxy divisions can be calculated as

$$\frac{q_E}{q_T} = \frac{q_E}{q_E + q_C} = \frac{1}{1 + (q_C / q_E)}, \qquad \text{(S6)}$$

where q_C / q_E is evaluated as

$$\frac{q_C}{q_E} = \frac{(T_B - T_{sat})/(R^C_{cond} + R^C_{conv})}{(T_B - T_{sat})/(R^E_{cond} + R^E_{conv})} = \frac{R^E_{cond} + R^E_{conv}}{R^C_{cond} + R^C_{conv}} . \qquad \text{(S7)}$$

Combining equations S5 and S7 yields

$$\frac{q_E}{q_T} = \left[1 + \frac{W_C}{W_E} \left(\frac{1 + hD/k_E}{1 + hD/k_C} \right) \right]^{-1} . \qquad \text{(S8)}$$

Additionally, the ratio of the wall superheat above the epoxy to the superheat above the copper can also be solved for as

$$\frac{\Delta T_{sat,E}}{\Delta T_{sat,C}} = \frac{T_E - T_{sat}}{T_C - T_{sat}} = \frac{q_E \, R^E_{conv}}{q_C \, R^C_{conv}}, \qquad \text{(S9)}$$

which reduces to

$$\frac{\Delta T_{sat,E}}{\Delta T_{sat,C}} = \frac{1 + hD/k_C}{1 + hD/k_E} . \qquad \text{(S10)}$$

Evaluation of equations S8 and S10 provides a first order estimate of the magnitude of the heat transfer rate through the epoxy divisions, as well as the ability of the bi-conductive surfaces to suppress the wall superheat above the epoxy divisions. It can be shown that during the nucleate boiling process, the percentage of heat transferred across the epoxy division is extremely small. Evaluating equation S8 using the experimentally measured heat transfer coefficients during nucleate boiling, h = 50–210 kW/m^{2}K (Figure 3), results in q_E / q_T = 0.2% - 2% for the highest performing surfaces (P = 3.7-1.8 mm). For the lower performing surfaces (P = 1.39-0.69 mm) this increases to q_E / q_T = 0.8% - 5%. Similarly, by evaluating equation S10 it can be shown that the ratio of the superheats between the epoxy and fluid to the copper and fluid is $\Delta T_{sat,E} / \Delta T_{sat,C}$ = 1.9% - 6.7%, over the same range of h.

Prior to the onset of nucleate boiling, the measured heat transfer coefficients reach values of $h \sim 5$ kW/m^{2}K. During this convection-dominated region, the lower heat transfer coefficients result in higher convective resistances that become comparable to the conductive resistances in the epoxy. Because of this, the percentage of heat transfer over the epoxy increases to q_E / q_T = 5% - 11% for the highest performing surfaces (P = 3.7-1.8 mm), and q_E / q_T = 15% - 24% for the lower performing surfaces (P = 1.39-0.69 mm), with $\Delta T_{sat,E} / \Delta T_{sat,C}$ = 41%. As discussed in the manuscript, this ~ 60% reduction in wall superheat over the epoxy divisions is sufficient to produce unstable convective flows and promote the preferential nucleation of vapor bubbles near the center of the copper segments.
